# Barriers to enrolment of clients into community client led anti-retroviral therapy delivery (CCLAD) in selected health facilities in Kasese district, Uganda

**DOI:** 10.4314/ahs.v24i1.7

**Published:** 2024-03

**Authors:** Kizito Omona, Alex Bwogi Kanyerezi

**Affiliations:** 1 Faculty of Health Sciences, Uganda Martyrs University, Kampala; 2 Department of Health and community Systems strengthening, Baylor-Uganda, Kampala

**Keywords:** Community client led anti-retroviral therapy delivery (CCLAD), Enrolment, Health care and public health

## Abstract

**Background:**

Community Client Led Anti-retroviral therapy Delivery (CCLAD) Model has been associated with increased community participation and ownership, which leads to better treatment outcomes with reduced workload and increased client satisfaction of health services.

**Aim:**

To explore the barriers to enrolment of eligible clients into CCLAD in selected health facilities in Kasese District, Uganda.

**Materials & methods:**

Analytical cross-sectional study utilizing mixed method approach was conducted among 384 PLWHIV attending public health facilities of Kasese District. Sampling was done by simple random sampling method. Data was collected using researcher-administered questionnaire method and interview guide.

**Results:**

Most of the respondents 253(65.9%) had not yet enrolled into CCLAD. This was due to some client-related factors such as non-disclosure of HIV sero-status (p=0.040), person to whom HIV sero-status was disclosed to (*p*=0.009), not having ever heard about CCLAD (*p*=0.000), incorrect description of CCLAD (*p*=0.000), limited knowledge of advantages of CCLAD (*p*=0.000) or disadvantages of CCLAD (*p*=0.003). Other barriers were; failure to have access to organizations or groups that support PLWHIV to get treatment (*p*=0.025) and duration of ART refills [AOR=1.637, 95% CI (0.820 – 3.270)].

**Conclusion:**

Adoption of CCLAD model among PLWHIV in Kasese District is still low.

## Introduction

### Background to the study

Community Client Led ART Delivery (CCLAD) are psychosocial community Anti-retroviral therapy (ART) groups comprising of stable clients living in the same community or locality. The CCLAD group members take turns to pick up ARVs at the health facility and distribute them among the other group members in the community 1. CCLAD is one of the five approved differentiated HIV treatment models in Uganda and that these models are meant to reduce pressure on overburdened health systems and to improve the quality of HIV care. According to the Joint United Nations Programme on HIV/AIDS (UNAIDS), the advent of antiretroviral therapy (ART) led to tremendous improvement in the quality of life of persons living with Human Immune Virus (PLWHIV), and greatly resulted in declines in the acquired immunodeficiency syndrome (AIDS) and associated morbidity and mortality rates 2. However, these could only be possible with sustained use and adherence to ART regimens, which often became hard in some communities due to challenges that make access to ART hard. Community Client Led ART Delivery (CCLAD), which is part of the community-based ART delivery model is community-based psychosocial intervention in which stable ART clients participate in the delivery of ART to other persons living with HIV/AIDS in their localities or communities 3. Globally, of the 38 million people estimated to be living with HIV globally, approximately 62% are on life-saving antiretroviral therapy (ART) and 53% are virally suppressed 4. Detectable viral load increases HIV-associated morbidity and mortality 5, and increases HIV transmission 6. Standard clinic-based delivery of ART, including a growing number of streamlined delivery models for stable patients who have suppressed viral load after 12 months of treatment, have successfully expanded ART coverage globally 7. However, in some settings, people with detectable viral load often have challenges of continuous access to ART due to a number of challenges such as transportation challenges to access services, thereby hindering the realisation of UNAIDS 90-90-90 targets of 2020 and 95-95-95 targets goals for 2030^2^,^4^. For example, there was a study about the current status toward the 90-90-90 UNAIDS targets and factors associated with HIV viral load suppression in Kediri City, Indonesia 8. The progress toward the 90-90-90 UNAIDS target was at 6.4%, 74.9%, 9.9%, respectively and the time taken by the HIV-positive patient to start ART treatment from the time of confirmation of HIV positive status (AOR= 83.191, CI: 1.617–4280.115) and decrease in body weight of the patient (AOR=29.636, CI: 1.193– 736.167) significantly influenced viral load suppression. Patients who took more than 1 year to start ART treatment were more likely (p<0.05, odds ratio = 83.191 > 1, 95% CI: 1.617–4280.115) to have a viral load non-suppression as compared to the patients who started ART treatment in less than 1 month 3,8. This is an indicator of the need for early initiation into ART care, and CCLAD model is one of the innovations that have proved to be effective in promoting linkage into ART care thereby leading to the realization of the second and third target under the 95-95-95 cascade 4.

In the generalized epidemic setting in southern and eastern Africa, the overall rate of viral suppression was 54% in South Africa and 64% in Uganda 2. This further indicates the great deal of work that has to be done in promoting and maintenance of PLWHIV into ART care. Client related barriers to care, such as missed wages, transport costs, and long waiting times for clinic visits and ART refills, are associated with detectable viral load 9. These barriers are amplified among certain groups of people, such as those living with disabilities, those whose businesses involved a lot of travel, domestic workers, those still in denial, those suffering from stigma and segregative gender norms and others. Due to their unique challenges, they 10. For example, Maughan-Brown et al.^11^ reported that linkage into care was negatively associated with treatment readiness (aOR: 2.97, 95% CI 1.05-8.34), weekly alcohol consumption (aOR: 0.35, 95% CI 0.12-0.98), and internalized stigma (aOR: 0.32, 95% CI 0.11-0.91) in South Africa. Such factors and challenges can be addressed through innovations in efficient service delivery, including expanding differentiated services to people with detectable viral load, particularly in order to achieve the UNAIDS 95-95-95 goal of 73% viral suppression among all people living with HIV 12. The CCLAD model was developed basing on lessons learned from chronic disease care in which providing more responsibilities to patients in the care of their chronic disease was found to be effective in limiting morbidity and mortality associated with some chronic diseases. In the CCLAD model, patients who are stable on ART participate in ART provision and peer support to their peers in same communities. The CCLAD group members take turns to pick up ARVs at the health facility and distribute them among the other group members in the community. They manage their own health and act with the support of Health community workers (HCWs). The CCLAD group members share experiences about living positive with HIV, and are empowered to offer and receive peer psychosocial support and follow-up 13. In CCLAD model, peer-led ART refill groups have demonstrated commendable success in four Sub-Saharan African countries, including Malawi, groups in South Africa, Democratic Republic of Congo (DRC), and Mozambique 1.

These countries are Malawi, South Africa, DRC and Mozambique. Peer-led ART refill groups lightened the burden for both patients and health system-reduced clinic attendance. It increased the retention in care and improved viral suppression. Retention in care was high - 94% at 36 months in Malawi, 89% at 12 months in DRC, 97% at 40 months in South Africa, and 92% at 48 months in Mozambique, respectively. Service provider costs were reported to be lower due to implementation of peerled ART refill models. In East Africa, a non-inferiority cluster-randomized pragmatic trial was done in Dar es Salaam, Tanzania. It indicated that an ARV community delivery model performed well. In Uganda, the Ministry of Health (MoH) okayed the use of community models to ensure continuity of ART, and during the COVID-19 period, to ensure accelerated decongestion of health facilities to minimize transmission of COVID-19^14^. The AIDS Support Organization (TASO), developed community-based ART delivery program beginning in 2006 in which ART care and treatment is delivered to consenting, stable patients at a pre-identified, community-based site 13,15. With the success of this model, TASO piloted the CCLAD model which further eased access to ART among HIV-positive persons in communities who don't have the ease of access to ART at health facilities or set community ART delivery points. However, in some communities, including those Kasese District, the CCLAD model hasn't yet proved to be effective in promoting adherence to ART, even after more than three years of its implementation in the district. This study sought to explore the barriers to enrolment of clients into community client led antiretroviral therapy delivery in selected health facilities in Kasese District.

### Aim of study

To explore the barriers to enrolment of PLWHIV into community client led antiretroviral therapy delivery at selected health facilities in Kasese District.

### Study objectives

The study was guided by the following specific objectives;

**1.** To determine the client-related barriers to the enrolment of PLWHIV into community client led ART delivery (CCLAD) at selected health facilities in Kasese District.

**2.** To find out the community-related barriers to the enrolment of PLWHIV into community client led ART delivery (CCLAD) at selected health facilities in Kasese District.

**3.** To ascertain the health facility-related barriers to the enrolment of PLWHIV into community client led ART delivery (CCLAD) at selected health facilities in Kasese District.

## Materials & methods

### Study design

The study utilized the analytical cross-sectional design with mixed method approach, specifically, Convergent parallel mixed method approach where quantitative and qualitative data were collected at the same time but analyzed separately.

### Study area

This study was conducted from selected public health facilities in Kasese District. The district is located in Western Uganda, along the equator. Kasese District is bordered by Kabarole District to the north, Kamwenge District and Kitagwenda District to the east, Rubirizi District to the south, and the Democratic Republic of the Congo to the west. The district headquarters at Kasese are located approximately 359 kilometres (223 mi), by road, west of Kampala, Uganda's capital and largest city. There are several health facilities in this district, including a district general hospital (public), a private-not-for profit hospital, two public health centre IVs, one private-not-for profit health centre IV, seven public health centre IIIs, two private-not-for profit health centre IIIs, and nine public health centres IIs, all of which provide HIV testing and ART services. However, the study was conducted among only public health facilities because they are the ones that implement the CCLAD model. This district was selected for this study because there are a number of CCLAD groups but with poor enrolment of PLWHIV into these groups.

### Study population

The study targeted people living with HIV/AIDS (PLWHIV) attending the selected public health facilities in Kasese District and key informant health workers. Health Facility ART in-charges (1 per facility) constituted the key informants.

### Eligibility criteria for recruitment in to the study

Both male and female PLWHIV were included in this study provided they: (1) were residents of Kasese District, (2) were on ART and (3) consented in writing to participate in the study, and (4) were least 18 years of age since this is the age of consent in Uganda. ART incharges who were actively involved in CCLAD were also included.

### Sample size determination

This sample size for this study was determined by the Kish (1968) formula 16 for cross-sectional studies:

$N=\frac{Z^{2}P(1-P)}{\delta 2}$

Where:

N= Required Sample Size

Z = Standard normal deviate at 95% confidence interval corresponding to 1.96

δ = Absolute error between the estimated and true population proportion (5%)

P = Population proportion of population with the factor under study. This was taken to be 50% (P = 0.50) since the proportion of PLWHIV on ART was not known in the study area.

Substituting in the formula:

$N=\frac{1.96\;2\;x\;0.5(1-051)}{(0.05)2}$

N= 384.16

However, a sample size of 384 was used for the quantitative aspect of the study. Six (6) key informant active ART in-charges were also recruited for qualitative investigation.

### Sampling technique

This was done at different levels, including selection of health facilities, and selection of the study participants. There are several health facilities in Kasese District which provide ART. However, in order to ensure unbiased representation, six of them were randomly selected to participate in the study. The names of the different health facilities were written on different pieces of paper of the same size, colour, texture and shape. The papers were placed in a small box and shaken. A health worker was asked to pick a piece of paper from the box without returning. This process was repeated five times until six pieces of paper had been picked. The names on the picked pieces of paper were those of the health facilities that had been selected to participate in the study.

Since six health facilities were participating in the study yet the sample size is 384, 64 PLWHIV were selected to take part in the study per facility. This was also done by random sampling to allow all PLWHIV equal opportunity of being selected to participate in the study. This was done over a period of four days, selecting 16 participants per day per facility, and it was done using the facility daily attendance register as the sampling frame. The names of PLWHIV available on the sampling day (according to the patient register by 10:00 AM) were each written on small pieces of paper of the same size, colour and texture. The papers were placed in a small box and thoroughly mixed. A health worker was asked to pick a piece of paper from the box without returning. This process was repeated until sixteen pieces of paper were picked per facility. The names of the picked piece of paper represented the PLWHIV who had been selected to participate in the study. Therefore, those who met the study inclusion criteria were considered for the study. If some of the selected PLWHIV were not eligible to participate in the study in line with the inclusion criteria, a replacement was done in the same process as described above. Six (6) ART in-charges were purposively selected for qualitative investigation.

### Study variables

The dependent variable for this study was enrolment into CCLAD. The independent variables were; the health facility-related barriers, the community-related barriers and client-related barriers to enrolment into CCLAD.

Community Client Led ART Delivery (CCLAD) are psychosocial community ART groups comprising of stable clients living in the same community/locality. The CCLAD group members take turns to pick up ARVs at the health facility and distribute them among the other group members in the community (Bemelmans, et al., 2014). In this study, enrolment into CCLAD was defined as belonging to a CCLAD group. Table 1 below shows how the different variables were measured.

**Table 1:** Study variables and their measurement

No.	Variable	Measurement
**Dependent Variable**		
	Enrolment into CCLAD	Was measured through a “Yes” or “No” response depending on whether the person is enrolled into CCLAD or not

**Independent Variables**		
	**(1) Client-Related Barriers** ■ Sociodemographic factors (age, gender, marital status, etc ■ Awareness/ knowledge ■ HIV disclosure ■ Duration on ART	Was measured categorically in two or more categories as: - Age (less or equal to 20year olds; 21 – 30; 31 – 40, and above 40 years of age - Gender (male or female) - Marital status (married or not married) - Education level (Primary, ‘O’ level, ‘A’ level, tertiary) - Awareness (aware or not aware) - Knowledge (knowledgeable or not knowledgeable) - HIV status disclosure (disclosed [and person disclosed to] or not yet disclosed) - Duration on ART (Less or equal to two years; 2 – 5 years; more than five years)
	
	**(2) Community-Related Barriers** ■ Stigma and discrimination ■ Distance to health facility ■ Transport ■ Community HIV support organisations/groups ■ Financial support	Was measured categorically in two or more categories as: - Experiencing stigma and discrimination due to HIV or not - Distance of five or less kilometers to public health facility or not - Having transport means (and type of transport means) or not - Presence of community HIV support organisations/groups or not
	
	**(3) Health Facility-Related Barriers** ■ Availability of ART ■ Availability of packaging material ■ Encouragement by health workers to join CCLAD ■ Incorporation of the component of CCLAD in health education ■ Linkage into CCLADD by facility ■ Duration of ART refills ■ Availability of guidelines ■ Supervision	Was measured categorically in two or more categories as: - Availability of ART at the facility or not - Availability of packaging materials or not - Encouragement by health workers to join CCLAD or not - Incorporation of the component of CCLAD in health education or not - Linkage into CCLADD by facility or not - Duration of ART refills (monthly, two month or more than two months) - Availability of guidelines or not - Supervision of CCLAD or not

### Data collection tools and procedures

A standard questionnaire was designed and was used during the data collection exercise for the primary respondents. It was arranged in sections, with section A having questions for determining the client-related barriers to the enrolment into CCLAD. Section B had questions for exploring the community-related barriers, while Section C had questions for determining the health facility-related barriers to enrolment into CCLAD. The data collection tool had both open and closed-ended questions. Open-ended questions were used to obtained detailed answers while closed-ended questions were used to obtain short and specific responses. The tool was developed in English language but the questions were translated into the local language for ease of understanding by the respondents.

Data from key informants was collected using an interview guide which had only open-ended questions in order to allow the key informant to give as much details as the could in line with the study objectives.

### Data analysis

Quantitative data collected was cleaned and sorted, and entered into the computerized statistical package for social scientists (SPSS) version 20. All the areas of investigation univariate, bivariate and multivariate analysis as appropriate. Pearson's Chi-square statistic was run at bivariate and multivariate logistic regression analysis in order to ascertain the different client-related barriers, community-related barriers, and health facility-related barriers to the enrolment into CCLAD. The significant level for all statistical analyses was set at p≤ 0.05. Qualitative data collected was analyzed verbatim.

### Quality controls

Quality control measures included those for ensuring validity, reliability, and training of research assistants.

### Validity

The questionnaire was given to two research experts who gauged the different components of the questionnaire to determine if they were relevant for achieving the research objectives. The research experts rated each component with 0, 1 or 2 scores depending on how they deemed them fit. Thus, the content validity index was computed as follows:

Content Validity Index


$$\text{Content}\;\text{Validity}\;\text{Index}=\frac{\text{Number}\;\text{of}\;\text{items}\;\text{declared}\;\text{valid}\;\text{by}\;\text{the}\;\text{research}\;\text{experts}}{\text{Total}\;\text{number}\;\text{of}\;\text{items}\;\text{on}\;\text{the}\;\text{instrument}}$$

Basing on the expert's analysis, an average content validity index score of 0.9 was obtained, higher than the 0.6 score recommended by Amin 17.

### Reliability

This was aimed at ensuring that the data collection tool was understandable by the study participants in order to enable them give commensurate information. As such, reliability was determined by pre-testing the data collection tool on fifteen PLWHIV at one health facility in that was not part of this study. The aim was to assess whether the questions were easy to understand. Adjustments in the questions was done as appropriate.

### Ethical considerations

Ethical considerations included the approval of the research protocols by the faculty of health sciences at Uganda Martyrs University and administrative clearance sought from relevant authorities in Kasese district, including the District Health Officer (DHO). Voluntary informed written consent was obtained from all the study participants. The principle for confidentiality was observed. Data collected was anonymized.

## Results

### Socio-demographic characteristics of respondents

The study was conducted among 384 PLWHIV attending the selected public health facilities in Kasese District (see table 2) and 6 key informants to find out the barriers to enrolment of PLWHIV into CCLAD. The key informants where in-charges of health facilities, four of whom were male and two females. They were serially coded as 1, 2, 3, 4, 5 & 6 respectively to conceal their identities and aid in qualitative analysis of their data. The socio-demographic characteristics of the 384 respondents are shown in table 2.

**Table 2:** Socio-demographic characteristics of the respondents

Characteristic			Frequency (n = 384)	Percent
**Age (years)**				
		Less or equal to 20	32	8.3%
		21-30	111	28.9%
		31-40	127	33.1%
		Above 40	114	29.7%

**Gender**				
		Male	146	38.0%
		Female	238	62.0%

**Marital status**				
		Married	246	64.1%
		Not married	138	35.9%

**Highest level of education**				
		Primary	208	54.2%
		Post-primary	156	40.6%
		Tertiary	20	5.2%

**Occupation**				
		None	68	17.7%
		Farmer/peasant	169	44.0%
		Small-scale business	113	29.4%
		Professional	34	8.9%

**Average monthly income (USH.)**				
		Less than UGX 50.000/=	105	27.3%
		UGX50.000/= to UGX100.000/=	171	44.5%
		More than UGX100.000/=	108	28.1%

**Disclosed HIV status**				
		Yes	342	89.1%
		No	42	10.9%

**Person disclosed HIV status to**				
		Close family member	155	40.4%
		Friend	150	39.1%
		Different person	37	9.6%
		N/A (Not disclosed)	42	10.9%

**Time spent on ART**				
		Less than 2 years	55	14.3%
		2-5 years	161	41.9%
		More than 5years	168	43.8%

Most of the respondents, 240(62.5%) were above the age of 30 years; majority, 238(62.0%) were female; majority, 246(64.1%) were married, and 208(54.2%) had primary level of education. Further, most of the respondents had either been on ART for more than 5 years (43.8%) or 2-5 years (41.9%) respectively. Most of them, 342(89.1%) had disclosed their HIV Sero-status, mainly to persons other than their close family members (59.6%).

### Client-related barriers to enrolment of PLWHIV into CCLAD

In addition to the socio-demographic factors, other client-related factors were considered as potential barriers to enrolment of PLWHIV into CCLAD. Binary and Multinomial logistic regression analysis was run and the results are shown in Table 3.

**Table 3:** Client-related barriers to enrolment of PLWHIV into CCLAD

Client-related Variables		Enrolled into CCLAD		Total	χ^2^	df	p-value	COR, 95% CI(L-U)	AOR, 95% CI (L-U)

		Yes	No						
**Age (years)**									
	Less than 20	7(1.8%)	25(6.5%)	32	8.579	3	**0.035** *	0.850(0.646 - 1.119)	0.663(0.213 - 2.070)
	20-30	29(7.6%)	82(21.4%)	111					
	31-40	52(13.5%)	75(19.5%)	127					
	Above 40	43(11.2%)	71(18.5%)	114					

**Gender**									
	Male	44(11.5%)	102(26.6%)	146	1.658	1	0.198	0.727(0.446 - 1.182)	0.707(0.427 - 1.170)
	Female	87(22.7%)	151(39.3%)	238					

**Marital status**									
	Married	93(24.2%)	153(39.8%)	246	4.147	1	**0.042** *	**1.470(0.891 - 2.426)** *	**1.553(0.919 - 2.624)** *
	Not married	38(9.9%)	100(26.0%)	138					

**Highest level of education**									
	Primary	71(18.5%)	137(35.7%)	208	6.841	2	0.077	0.936(0.688 - 1.274)	0.605(0.172 - 2.134)
	Post-primary	48(12.5%)	108(28.1%)	156					
	Tertiary	12(3.1%)	8(2.1%)	20					

**Occupation**									
	None	20(5.2%)	48(12.5%)	68	3.845	3	0.279	1.047(0.737 - 1.486)	1.042(0.286 - 3.790)
	Farmer/Peasant	60(15.6%)	109(28.4%)	169					
	Small-scale business	35(9.1%)	78(20.3%)	113					
	Professional	16(4.2%)	18(4.7%)	34					

**Average monthly income (UGX)**									
	Less than UGX 50.000/=	36(9.4%)	69(18.0%)	105	0.99	1	0.320	0.956(0.686 - 1.334)	0.897(0.450 - 1.788)
	UGX50.000/= to UGX100.000/=	54(14.1%)	117(30.5%)	171					
	More than UGX100.000/=	41(10.7%)	67(17.4%)	108					

**Disclosed HIV status**									
	Yes	122(31.8%)	220(57.3%)	342	3.377	1	0.066	**1.315(0.539 - 3.210)** *	**1.236(0.478 - 3.197)** *
	No	9(2.3%)	33(8.6%)	42					

**Person disclosed HIV status to**									
	Close family member	67(17.4%)	88(22.9%)	155	11.539	3	**0.009** *	**1.392(1.072-1.807)** *	**2.630(1.007 - 6.868)** *
	Friend	44(11.5%)	106(27.6%)	150					**1.746(0.658 - 4.632)** *
	Different person	12(3.1%)	25(6.5%)	37					**1.347(0.422 - 4.301)** *
	N/A (Not disclosed)	8(2.1%)	34(8.9%)	42					

**Time spent on ART**									
	Less than 2 years	16(4.2%)	39(10.2%)	55	5.378	2	0.068	0.872(0.605 - 1.256) *	0.883(0.403 - 1.934)
	2-5 years	47(12.2%)	114(29.7%)	161					
	More than 5 years	68(17.7%)	100(26.0%)	168					

**Ever heard about CCLAD**									
	Yes	128(33.3%)	217(56.5%)	345	13.483	1	**0.000** *	**9.376(1.914-45.922)** *	1.240(0.008 - 193.328)
	No	3(0.8%)	36(9.4%)	39					

**Respondent correctly describes CCLAD**									
	Platform for client groups comprising of stable ART clients living in the same community to access ART in the community	119(31.0%)	183(47.7%)	302	17.604	1	**0.000** *	**2.556(1.108-5.898)** *	**1.989(0.826-4.789)** *
	Don't Know/Never heard of CCLAD)	12(3.1%)	70(18.2%)	82					

**Respondent knows advantages of CCLAD as specified**									
	Time saving	77(20.1%)	155(40.4%)	232	26.581	3	**0.000** *	**0.598(0.427-0.837)** *	**2.166(0.014 - 339.56)** *
	Helps each other	27(7.0%)	48(12.5%)	75					
	Saves money	24(6.3%)	14(3.6%)	38					
	Doesn't know	3(0.8%)	36(9.4%)	39					

**Respondent knows the disadvantages of CCLAD as specified**									
	Only relatively good on treatment	9(2.3%)	13(3.4%)	22	11.419	1	**0.003** *	**1.465(0.916 -2.035)** *	**1.450(0.501 - 4.200)** *
	Worried about confidentiality	67(17.4%)	88(22.9%)	155					
	Doesn't know	55(14.3%)	152(39.6%)	207					

* Denotes significant finding, UGX= Ugandan Shillings, N/A = Not applicable, CI=Confident Interval, p-value =Probability value, COR=Crude Odd Ratio, AOR=Adjusted Odd Ratio, L=Lower boundary, U=Upper boundary

As shown in table 3, the only client-related variables that were found to be associated barriers to enrolment of PLWHIV into CCLAD were: age (p = 0.035), marital status (p = 0.042), education level (p-value = 0.007), disclosure of HIV sero-status (p-value = 0.040), person to whomIV sero-status was disclosed to (p = 0.009), having ever heard about CCLAD (p = 0.000), correct description of CCLAD (p = 0.000), knowledge of advantages of CCLAD (p = 0.000) and knowledge of disadvantages of CCLAD (p = 0.003).

At both bivariate and multivariate analysis, respondents who were married were 1.5 times more likely to enrol into CCLAD [COR=1.470; 95% CI (0.891 - 2.426) and AOR = 1.553; 95% CI (0.919 – 2.624)]. Respondents who disclosed their HIV status were 1.3 times more like to enrol into CCLAD [COR=1.315; 95% CI (0.539 - 3.210)]; hence failure to disclose was a barrier. Respondents who ever heard about CCLAD were 9 times more likely to enrol into CCLAD [COR=9.376; 95% CI (1.914 – 45.922)] than those who didn't hear about it. Not knowing how to describe CCLAD was a big barrier to enrolment as respondents who could correctly describe CCLAD where 2.6 [COR=2.556; 95% CI (1.108 - 5.898)] times and 2 times [AOR=1.989; 95% CI (0.826-4.789)] more likely to enrol into CCLAD at bivariate and multivariate level of analysis respectively. Poor knowledge of the advantages of CCLAD was found to be a barrier to enrolment, as those who knew the advantages were 2 times more likely to enrol [AOR=2.166; 95% CI (0.014 -339.56)]. Similarly, not knowing the disadvantages of CCLAD was also a barrier to enrolment, as those who knew the disadvantages were 1.5 times more likely to enrol into CCLAD [AOR= 1.450; 95% CI (0.501 - 4.200)].

Findings from key informants (KI) also pointed to some client-related factors that present as barriers to enrolment into CCLAD. For example, KI related lack of enrolment into CCLAD to none-disclosure of HIV status disclosure, as can be observed in their quotes below:

*“[…] none-disclosure of HIV sero-status is the main hinderance to enrolment into CCLAD […]”* **- Key Informants 2 & 6**

*“[…] some persons living with HIV fear disclosing their HIV sero-status which hinders their participation in community client led ART delivery […]” –* **Key Informants 1, 3 & 4**

*“[…] my observation is that PLWHIV who don't disclose their HIV sero-status find it hard to join CCLAD groups… they would rather walk long distance to pick their ART refills from the health facilities than pick it from a peer within their communities […]”* **- Key Informant 5**

Further, analysis of finding indicated that Key informants blamed the lack of enrolment into CCLAD to not having clear understanding about this model. This can be observed in the quotes below:

*“[…] some of the persons living with HIV still don't understand the CCLAD model, despite health workers undertaking to often health educates about this model. This hinders their enrolment into CCLAD groups […]” –* **Key Informant 2**

### Community-related barriers to enrolment of PLWHIV into CCLAD

A number of community-related variables were considered in this study, including: stigma or discrimination, distance from home to the health facility, ease of access to transport means, etc. Bivariate and multivariate analysis was run and the results are show in table 4.

**Table 4:** community-related barriers to enrolment of PLWHIV into CCLAD

Community-related Variables		Enrolled Into CCLAD		Total	χ^2^	df	p-value	COR, 95% CI (L-U)	AOR, 95% CI (L-U)
	
		Yes	No						
**Ever experienced stigma and discrimination due to HIV**									
	Yes	28(7.3%)	59(15.4%)	87	0.187	1	0.666	0.860(0.508 - 1.456)	0.620(0.187 – 2.056)
	No	103(26.8%)	194(50.5%)	297					

**Forms of stigma and discrimination ever experienced**									
	Denied access to social services	18(4.7%)	25(6.5%)	43	2.321	2	0.313	**1.425(0.890 - 2.281)** *	**1.647(0.623 – 4.355)** *
	Rebuked by family members	11(2.9%)	31(8.1%)	42					
	N/A (No stigma)	102(26.6%)	197(51.3%)	299					

**Distance from home to the health facility**									
	Less or equal to 5 kilometres	77(20.1%)	144(37.5%)	221	0.122	1	0.726	1.029(0.666 – 1.590)	1.084(0.683 – 1.720)
	More than 5 kilometres	54(14.1%)	109(28.4%)	163					

**Means used to access the health facility**									
	Bicycle	32(8.3%)	80(20.8%)	112	2.205	3	0.531	0.857(0.688 – 1.066)	0.643(0.312 – 1.326)
	Motor-cycle	56(14.6%)	100(26.0%)	156					
	Vehicle	22(5.7%)	37(9.6%)	59					
	Foot (Short distance)	21(5.5%)	36(9.4%)	57					

**Easy to access transport means to the health facility**									
	Yes	70(18.2%)	136(35.4%)	206	0.004	1	0.952	0.973(0.627 - 1.512)	0.864(0.544 - 1.373)
	No	61(15.9%)	117(30.5%)	178					

**Has access to organizations or groups that support persons living with HIV to get treatment**									
	Yes	81(21.1%)	126(32.8%)	207	5.027	1	**0.025**	**1.758(0.937 - 3.297)** *	0.682(0.070 - 6.617)
	No	50(13.0%)	127(33.1%)	177					

**Organization that supports you with HIV treatment**									
	None	50(13.0%)	127(33.1%)	177	10.381	5	0.065	1.023(0.862 - 1.213)	1.363(0.549 - 3.385)
	TASO	14(3.6%)	15(3.9%)	29					
	Baylor	19(4.9%)	32(8.3%)	51					
	Good Hope	3(0.8%)	4(1.0%)	7					
	Yeyi	6(1.6%)	20(5.2%)	26					
	Others	38(9.9%)	51(13.3%)	89					

* Denotes significant finding, N/A = Not applicable, CI = Confident Interval, p-value =Probability value, COR=Crude Odd Ratio, AOR=Adjusted Odd Ratio, L=Lower boundary, U=Upper boundary

According to study results in Table 4, it was found that failure to have access to organizations or groups that support persons living with HIV to get treatment was a barrier as respondents who had access to such organization (p=0.025). Having such organization or group makes one 1.8 times more likely to enrol in to CCLAD [COR=1.758, 95% CI (0.937 – 3.297)] as oppose to those who didn't have. Similarly, respondents who experienced different forms of stigmatization or discrimination were 1.6 times more likely to enrol in to CCLAD [AOR=1.647, 95% CI (0.623 – 4.355)].

Findings from key informants (KI) also pointed to some community-related factors that presented as barriers to enrolment into CCLAD. For example, KI attributed the lack of enrolment into CCLAD partly on existence of other organisations supporting PLWHIV, as can be observed in their quotes below:

*“[…] some communities are supported by other implementing partners (Non-Governmental Organisations) that deliver services (ART) directly to their clients in the communities. Those clients fail to join CCLAD groups as they don't see the need since the medicines are delivered close to them […]” –* **Key Informant 3**

*“[…] lack of enrolment into CCLAD is more common in communities with other organisations that support persons living with HIV […]” –* **Key Informants 1 and 3**

### Health facility-related barriers to enrolment of PLWHIVA into CCLAD

To determine the health facility-related barriers to enrolment into CCLAD, bivariate and multivariate regression was run and the results shown in table 5. The variables considered were: ease of access of ART services, availability of ART medicines, duration of ART refills, health workers incorporating component of CCLAD in health education talks, health worker encourage them to join CCLAD groups, health workers linking them to CCLAD groups, health facilities having guidelines on CCLAD model, health workers supervising CCLAD groups, and health workers doing enough to promote CCLAD model.

**Table 5:** Health facility-related barriers to enrolment of PLWHIV into CCLAD

Health Facility-Related Variables		Enrolled Into CCLAD		Total	χ^2^	df	p-value	COR, 95% CI (L-U)	AOR, 95% CI (L-U)

		Yes	No						
**Respondent Often finds it easy to access ART services at the health facility**									
	Yes	128(33.3%)	244(63.5%)	372	0.458	1	0.499	2.366(0.460 –12.167)*	2.497(0.473 – 13.183) *
	No	3(0.8%)	9(2.3%)	12					

**ART medicines are available**									
	Yes	115(29.9%)	241(62.8%)	356	7.126	1	0.008*	0.139(0.039 - 0.493)	0.131(0.036 - 0.480)
	No	16(4.2%)	12(3.1%)	28					

**Duration of ART Refills**									
	Monthly	32(8.3%)	40(10.4%)	72	5.956	2	0.051	1.178(0.847-1.638)	1.637(0.820 – 3.270) *
	Two months	29(7.6%)	79(20.6%)	108					
	More than two months	70(18.2%)	134(34.9%)	204					

**Health workers often incorporate component of CCLAD in the health education skills**									
	Yes	97(25.3%)	149(38.8%)	246	8.608	1	0.003*	0.690(0.360 – 1.3233)	0.709(0.369 - 1.361)
	No	34(8.9%)	104(27.1%)	138					

**Health workers encouraged me to join a CCLAD group**									
	Yes	118(30.7%)	164(42.7%)	282	28.219	1	0.000*	1.499(0.552 - 4.065)*	1.370(0.501 - 3.746) *
	No	13(3.4%)	89(23.2%)	102					

**Health worker linked me to a CCLAD group**									
	Yes	108(28.1%)	83(21.6%)	191	85.062	1	0.000*	6.695(3.416 -13.120)*	6.563(3.329 - 12.938) *
	No	23(6.0%)	170(44.3%)	193					

**Health facility has guidelines on CCLAD model**									
	Yes	100(26.0%)	155(40.4%)	255	8.788	1	0.003*	0.819(0.435 -1.542)	0.848(0.449 - 1.602)
	No	31(8.1%)	98(25.5%)	129					

**Health workers supervise our CCLAD groups**									
	Yes	112(29.2%)	134(34.9%)	246	39.676	1	0.000*	2.294(1.048 - 5.024)*	2.361(1.075 - 5.183) *
	No	19(4.9%)	119(31.0%)	138					

**Health workers doing enough to promote CCLAD model**									
	Yes	103(26.8%)	146(38.0%)	249	16.567	1	0.000*	0.938(0.340 - 2.588)	1.216(0.259 - 5.717)
	No	28(7.3%)	107(27.9%)	135					

**How health workers are doing enough to promote CCLAD**									
	Encourage formation of more CCLAD groups	59(15.4%)	74(19.3%)	133	19.633	2	0.000*	1.157(0.699 - 1.915)	1.094(0.242 - 4.947)
	Ever talking to community about CCLAD	41(10.7%)	61(15.9%)	102					
	Not doing enough	31(8.1%)	118(30.7%)	149					

It was found that the significant barriers to enrolment into CCLAD were as follows: availability of ART medicines (p = 0.008), duration of the ART refills [AOR=1.637, 95% CI (0.820 – 3.270)], health workers incorporating component of CCLAD in health education skills (p = 0.003), health workers encouraging them to join CCLAD groups (p = 0.000), health workers linking them to CCLAD groups (p = 0.000), health facilities having guidelines on CCLAD model (p = 0.003), health workers supervising CCLAD groups (p = 0.000), health workers doing enough to promote the CCLAD model (p = 0.000), and how health workers are doing enough to promote CCLAD (p = 0.003). Details are shown in table 5. Respondents who had ease in accessing ART were 2.5 times more likely to enrol into CCLAD [AOR=2.497, 95% CI (0.473 – 13.183]. Health workers encouraging client to join a CCLAD group was 1.5 times more impactful of enrolment in to CCLAD [COR= 1.499, 95% CI (0.552 - 4.065)]. Again, health workers linking clients directly to CCLAD [AOR=6.563, 95% CI (3.329 -12.938)] and supervising CCLAD groups [AOR=2.361, 95% CI (1.075 - 5.183)] were 6.5 times and 2.4 times more impactful in enrolment to CCLAD as shown in table 5.

Findings from key informants (KI) also pointed to some of the health facility-related factors that presented as barriers to enrolment into CCLAD. For example, Key Informants attributed the lack of enrolment into CCLAD to unavailability of medicines, as can be observed in their quotes below:

*“[…] some persons living with HIV stay close to health facilities where they can pick their medicines at any time since those medicines are readily available. This affects whether or not they should enrol into CCLAD […]” –* **Key Informants 4 and 6**

Further, not being linked to CCLAD groups by health workers was also highlighted by the KI as a barrier to enrolment into CCLAD, as can be observed in the quotes below:

*“[…] one of the main reasons for lack of enrolment into CCLAD is health workers not linking their clients to existing groups […]” –* **Key Informant 3**

*“[…] some health workers don't link persons living with HIV to CCLAD groups […], which negatively influences those persons interest in joining CCLAD groups […]” -* **Key Informants 2 and 5**

## Summary of results

The study found that majority of the PLWHIV, 253(65.9%) had not yet been enrolled into CCLAD. This was due to some client-related factors such as non-disclosure of HIV sero-status (p-value = 0.040), person to whom HIV sero-status was disclosed to (p = 0.009), not having ever heard about CCLAD (p = 0.000), incorrect description of CCLAD (p = 0.000), limited knowledge of advantages of CCLAD (p = 0.000) or disadvantages of CCLAD (p = 0.003). The other barriers to enrolment into CCLAD were community-related, such as failure to have access to organizations or groups that support persons living with HIV to get treatment was a barrier as respondents who had access to such organization (p=0.025). Having such organization or group makes one 1.8 times more likely to enrol in to CCLAD [COR=1.758, 95% CI (0.937 – 3.297)] as oppose to those who didn't have. Similarly, respondents who experienced different forms of stigmatization or discrimination were 1.6 times more likely to enrol in to CCLAD [AOR=1.647, 95% CI (0.623 – 4.355)]. Duration of the ART refills [AOR=1.637, 95% CI (0.820 – 3.270)], health workers incorporating component of CCLAD in health education skills (p = 0.003) also affected enrolment significantly, among others.

### Appendix I_Questionnaire

**Figure:**
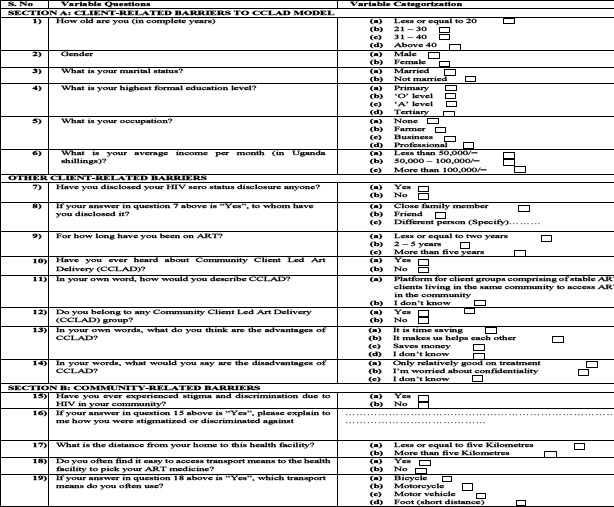

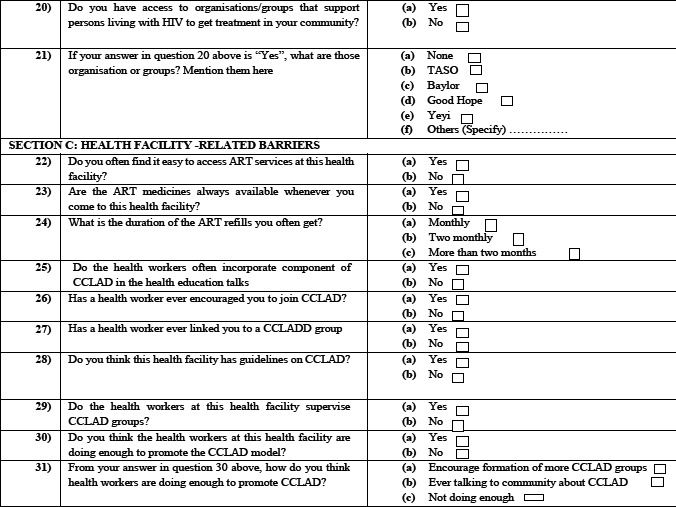


### Appendix II_Interview Guide for Key Informants

You are hereby requested to participate in this study by sharing your thoughts according to the research questions below:

1) What are the client-related barriers to the enrolment of persons living with HIV into community client led ART delivery at selected health facilities in Kasese District?
  ………………………………………………………………………………………..
2) What are the community-related barriers to the enrolment of persons living with HIV into community client led ART delivery at selected health facilities in Kasese District?
  ………………………………………………………………………………………..
3) What are the health facility-related barriers to the enrolment of persons living with HIV into community client led ART delivery at selected health facilities in Kasese District?
  ………………………………………………………………………………………..

## Discussion

### Client-related barriers to enrolment of PLWHIV into CCLAD

In this study, the client-related barriers to enrolment of PLWHIV into CCLAD were: disclosure of HIV status, persons disclosed HIV status to, and knowledge of description of CCLAD. Respondents who disclosed their HIV status were 1.3 times more like to enrol into CCLAD [COR=1.315; 95% CI (0.539 - 3.210)]. This, in essence, implies that those who hadn't declared their HIV sero-status were less likely to enrol into CCLAD groups. Probably the non-disclosure of HIV sero-status made them to fear to interact with other PLWHIV, which potentially hindered their enrolment into CCLAD groups for fear of interacting with others who were living with HIV. Fearing to disclose HIV sero-status raises concerns over ability of community led programs to influence good practices aimed at combating HIV/AIDS. This raises more worries about Uganda being able to meet the 95-95-95 HIV control by 2030 since non-disclosure of sero-status can breed new HIV infections. The finding on impact of non-disclosure of HIV sero-status is in line with the study at Mulago adult HIV clinic, which also found non-HIV status disclosures as being a barrier to enrolment into CCLAD, among other factors 3,18-20. Further, in line with HIV status disclosure, those who had disclosed their HIV sero-status to a different person other than a close family member were about 2.6 times more likely not to be enrolled into CCLAD. This can further be attributed to the fear to interact with other PLWHIV due to not liking to disclose the HIV status to them. However, this is somehow opposed in the study by Omona and Sabiti in Mulago 3. The difference in findings could be attributed to differences in study setting settings.

Again, in this current study, those who knew the correct description of CCLAD were more likely to be enrolled into CCLAD than those who didn't know the correct description of CCLAD. Probably knowing the description of CCLAD was associated with the benefits of community HIV support groups, which probably increased their confidence to belong to a CCLAD groups. On the other hand, not knowing the correct description of CCLAD probably meant that PLWHIV didn't have clear understanding about CCLAD, and therefore did not appreciate joining such groups. This finding is supported by Amuron et al. 21 in a study in Jinja which also highlighted that belonging to HIV community support groups was highest among those who had good knowledge about those groups, and lowest among those who didn't have good understanding about such groups. These findings imply that health workers and those involved in HIV advocacy and other related interventions should focus on increasing awareness about CCLAD so that PLWHIV embrace such supportive groups. Not doing this tantamount to jeopardizing intervention aimed at controlling HIV through the use of community-led support groups such as CCLAD.

### Community-related barriers to enrolment of PLWHIV into CCLAD

The community- related barriers to enrolment of PLWHIV into CCLAD were: absence of organisations that support PLWHIV to get treatment. Respondents who had such supportive organisations in their communities were 1.8 times more likely to enrol in to CCLAD [COR=1.758, 95% CI (0.937 – 3.297)] as oppose to those who didn't have. Probably the existence of organisation made them have the need of requiring support of their peers in obtaining their ART refills. This could have been the reason as to why those who had TASO, Baylor, and other organisations in their communities were more supported to enrol. These findings imply that for the success of CCLAD models, it is better that such community-based interventions are prioritised in areas with limited access to support, especially communities that don't have other partners who promote the same services. This is key for avoidance of duplication of services but also for better utilisation of the limited resources available for combating HIV/AIDS. The findings are supported by Long et al. 22 who pointed that peer-led ART delivery models are most likely to fail in communities where there exist other organizations with different motives. This is similar to documented experiences and perceptions on CCLADS Model from Patients' and Providers' Perspectives in South Western Uganda 23. Many other scholars found similar results related to discrimination 20.

### Health facility-related barriers to enrolment of PLWHIV into CCLAD

The health facility- related barriers to enrolment of PLWHIV into CCLAD were: unavailability of ART medicines, failure of health workers to link clients to CCLAD groups, and how they do it. Respondents who had ART medicines readily available were more likely to be enrolled into CCLAD than those who didn't (p=0.008). This finding is inconsistent with that of Decroo et al. 24 who also pointed out that ease of availability of ART medicines, mostly due to close proximity to health facilities acted as strong barrier to the success of CCLAD models.

In this present study, those who were linked by health workers to CCLAD groups were more likely to be enrolled into CCLAD than those who were not. Probably not linking them to CCLAD groups made them not to appreciate the relevance of being enrolled into CCLAD. Nonetheless, the findings imply that CCLAD model can only be effective if nearby all health facilities proactively link HIV clients to the CCLAD groups in their communities. This kind of linkage creates confidence, and is therefore, likely to be associated with client appreciation and acceptance to belong to CCLAD groups. This finding is supported by Kandasami et al. 25 who also pointed out the positive correlation between health worker encouragement and PLWHIV enrolling into CCLAD. Many other studies gave similar findings 20.

## Conclusion

The study found that while community-based ART programs have achieved remarkable results in expanding access to and adherence to ART in resource-poor settings, most PLWHIV had not yet been enrolled into CCLAD, despite this model being operational in the district. The study found the barriers to enrolment into CCLAD to be mainly due to client-related factors such as not disclosing the HIV status, and others. The other community-related barriers such as not having organisations in their communities that support PLWHIV to get treatment, as well as health facility-related barriers such as not being linked by health workers to CCLAD groups, and health workers not encouraging them to form CCLAD groups could doubly complicate the uptake of CCLAD.

## Recommendations

The authors recommend as follows;

**1.** ART Clinic Healthcare Workers should support PLWHIV to consider disclosing their HIV sero-status to their spouses and a few more family members since the lack of such disclosure is a hindrance to enrolment into CCLAD.

**2.** ART Clinic Healthcare Workers should consider giving sufficient information regarding CCLAD to all clients on ART. This will improve their understanding about this model and positively influence enrolment into CCLAD.

**3.** Ministry of health should provide sufficient guidance on how the CCLAD model can continue to work despite the presence of organisations that support PLWHIV in communities.

**4.** Kasese District Health Office should consider undertaking effective supervision of health workers in ART clinics to ensure they give information about CCLAD to all clients on ART. This will eventually encourage linkage of all clients to CCLAD groups in the communities.

## Limitations of the study

Data was obtained through self-reporting by the study participants which could have been subject to individual biases in the responses given by the participants. However, they were reassured, and their fears addressed to enable them to comfortably give the required information for the study. Sufficient explanation and help were made during the interview sessions.

## Authors' info

Dr. Omona Kizito is a Medical Doctor and Lecturer in the Faculty of Health Sciences of Uganda Martyrs University. He holds a PhD in Management (Healthcare Management), Master of Science in Health Services Management, Master of Science in Monitoring and Evaluation, Postgraduate Diploma in Project Planning and Management and Bachelor of Medicine and Bachelor of Surgery (MBChB) from Gulu University. He is a senior researcher in the areas of Public Health, Health Services Management, Child focused research.

Kanyerezi Bwogi Alex is currently working as a Community Systems Strengthening Officer with Baylor-Uganda in the Health and community Systems strengthening department. He holds a Bachelors in community psychology from Makerere University and accumulated training in Behaviour change and communication in HIV as well as Master of Public Health degree.

## Data sharing statement

Dataset related to this study is available and published^26^. It can be accessed from https://doi.org/10.7910/DVN/4QJTJK.
